# Curcumin reduces α-synuclein induced cytotoxicity in Parkinson's disease cell model

**DOI:** 10.1186/1471-2202-11-57

**Published:** 2010-04-30

**Authors:** Min S Wang, Shanta Boddapati, Sharareh Emadi, Michael R Sierks

**Affiliations:** 1 Department of Chemical Engineering, Arizona State University, Tempe, AZ 85287-6006 USA; 2 Department of Chemistry, University of Colorado Denver, Denver, CO 80217-3364 USA

## Abstract

**Background:**

Overexpression and abnormal accumulation of aggregated α-synuclein (αS) have been linked to Parkinson's disease (PD) and other synucleinopathies. αS can misfold and adopt a variety of morphologies but recent studies implicate oligomeric forms as the most cytotoxic species. Both genetic mutations and chronic exposure to neurotoxins increase αS aggregation and intracellular reactive oxygen species (ROS), leading to mitochondrial dysfunction and oxidative damage in PD cell models.

**Results:**

Here we show that curcumin can alleviate αS-induced toxicity, reduce ROS levels and protect cells against apoptosis. We also show that both intracellular overexpression of αS and extracellular addition of oligomeric αS increase ROS which induces apoptosis, suggesting that aggregated αS may induce similar toxic effects whether it is generated intra- or extracellulary.

**Conclusions:**

Since curcumin is a natural food pigment that can cross the blood brain barrier and has widespread medicinal uses, it has potential therapeutic value for treating PD and other neurodegenerative disorders.

## Background

Parkinson's disease (PD) affects 1% of the population over the age of 65 and is the second most common progressive neurodegenerative disorder after Alzheimer's disease (AD) [1,2]. The classical symptoms of PD include resting tremor, muscular rigidity and bradykinesia [2,3] resulting from the progressive loss of dopaminergic neurons in the *substantia nigra *region of the brain [3,4]. Intracellular inclusions known as Lewy bodies (LB) and Lewy neurites (LN), composed primarily of insoluble aggregates of ubiquitin and α-synuclein (αS), are neuropathological hallmarks of PD found in many regions of the brain and central nervous system (CNS) [4-6]. Point mutations and multiplication of the αS gene are associated with rare early onset familial forms of the disease, further implicating the role of αS in PD [7-10]. The increased degeneration of dopaminergic neurons in the *substantia nigra *of PD animal models correlates with increased levels of LBs and LNs in this region of the brain and strongly suggests that overexpression of αS selectively targets dopaminergic neurons [11-13]. While it is unclear why dopaminergic neurons are more susceptible to degeneration by αS, the oxidation of dopamine and exposure to neurotoxins such as rotenone [14,15] and 1-methyl-4-phenyl-1,2,3,6-tetrahydropyridine (MPTP) [16-19] generate excessive reactive oxygen species (ROS), promoting mitochondrial complex I dysfunction [15,20,21] and depleting glutathione levels [22,23] ultimately causing acute Parkinsonism in animal and cell models. In addition, overexpression of both wild type (WT) and mutant αS results in formation of cytoplasmic inclusions and degeneration of dopaminergic neurons in mouse and *Drosophila *models [11-13,24].

αS is a presynaptic protein expressed at synaptic terminals in the CNS [25,26]. While αS is a natively unfolded protein, the monomeric form can misfold and aggregate into larger oligomeric and fibrillar forms which are linked to the pathogenesis of PD. Recent studies have implicated small soluble oligomeric and protofibrillar forms of αS as the most neurotoxic species [27-30]. While previous studies provide good evidence for the intracellular toxicity of αS in PD, there is also evidence showing an extracellular component as well [27-29,31,32]. Monomeric and oligomeric forms of αS have been detected in blood plasma and cerebrospinal fluid of PD patients [27,31-33], and exposure to extracellular pre-aggreated αS induces cytotoxicity in primary mesencephalic neuron-glia and human neuroblastoma cell cultures [28,29,34,35].

Since generation of ROS has been correlated with onset of PD, anti-oxidants may have therapeutic value. Curcumin, a polyphenolic compound commonly used as food additives in Asian cuisine, has anti-oxidant properties and suppresses inflammatory responses of brain microglial cells [36-38]. Curcumin was also shown to have protective effects in neurodegenerative disease by either reducing inflammation and oxidative damage in AD [36-39], or by inhibiting protein misfolding and aggregation in Creutzfeld-Jakob disease [40] and PD [41,42].

Given these numerous beneficial properties, curcumin shows promise as a therapeutic agent for neurodegenerative diseases. We show that curcumin can provide protection against αS-induced cytotoxicity in SH-SY5Y neuroblastoma cells by decreasing cytotoxicity of aggregated αS, reducing intracellular ROS, inhibiting caspase-3 activation and ameliorating signs of apoptosis. We also show that either extracellular addition of oligomeric αS and intracellular overexpression of αS increases generation of intracellular ROS in SH-SY5Y cells and both have similar cytotoxic effects resulting in induced caspase-3 activity and apoptosis.

## Results

### Curcumin protects SH-SY5Y cells against extracellular αS-induced cytotoxicity

**Figure 1:**
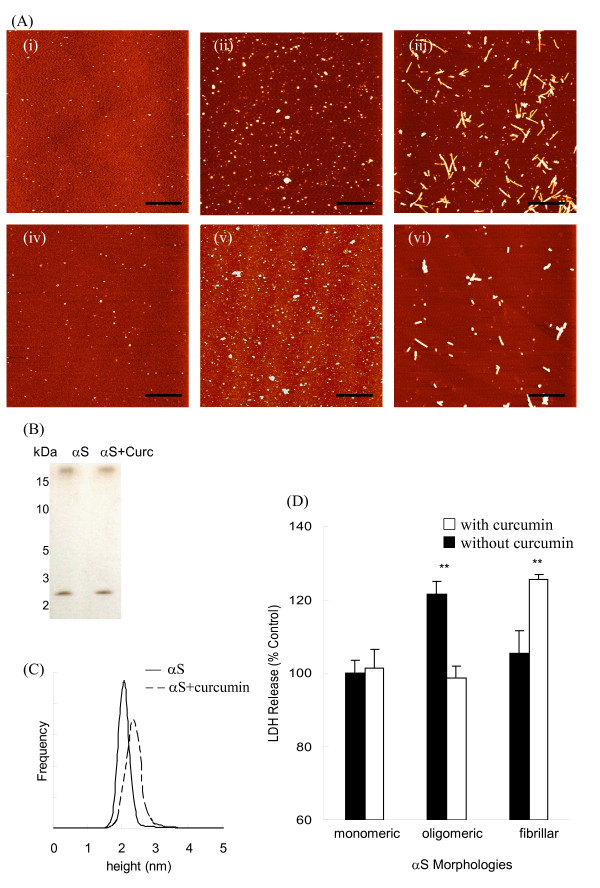
**Oligomeric αS induces cytotoxicity in SH-SY5Y cells**. Conformation and cytotoxicity of αS with and without curcumin addition observed by AFM imaging, PAGE and LDH assay. (A) AFM images of αS alone: (i) monomeric αS, (ii) pre-formed oligomeric αS and (iii) fibrillar αS; and αS co-incubated with curcumin for 2-h: (iv) monomeric αS, (v) pre-formed oligomeric αS and (vi) and fibrillar αS. Scale bar = 1 μm. (B) Pre-formed oligomeric αS samples, with and without curcumin were separated on a 10% Tris/Tricine native PAGE gel and analyzed using silver staining. (C) Height distribution of oligomeric αS samples. Particle heights of pre-formed oligomeric αS samples, with (--) and without curcumin (-) were analyzed using AFM and SPIP software. (D) LDH activity of SH-SY5Y cells incubated with different morphologies of αS with or without co-incubation with curcumin. LDH release was expressed as a percentage of the Tris control samples. Data was reported as mean ± SE, *n *= 4. **p < 0.01 compared with the untreated control samples.

Extracellular incubation of SH-SY5Y cells with oligomeric but not monomeric or fibrillar αS induced significant cytotoxicity (Fig. 1) in agreement with previous studies implicating oligomeric αS as the toxic species [27-30]. While co-incubation of curcumin does not alter the monomeric and pre-formed oligomeric αS morphologies, it does destabilize pre-formed αS fibrils (Fig. 1A, Additional file 1), consistent with previous results [41]. PAGE and AFM size distribution data also confirm that curcumin does not alter the molecular weight or size of the oligomeric αS species (Fig. 1B and 1C). Toxicity assays show that addition of curcumin significantly reduces the αS-induced toxicity induced by pre-formed oligomeric αS while co-incubation of curcumin with pre-formed αS fibrils shows a significant increase in toxicity (Fig. 1D). Co-incubation of curcumin with monomeric αS does not alter cytotoxicity (Fig. 1D) similar to incubation with curcumin and Tris buffer alone (Table 1). Toxicity studies of curcumin alone towards SH-SY5Y cells showed no toxic effects at concentrations below 5 μM (data not shown). Since only oligomeric αS aggregates induced toxicity in SH-SY5Y cells, subsequent experiments were performed with oligomeric αS to determine the protective effects of curcumin against αS.

**Table 1:** Curcumin neutralizes αS-induced cytotoxicity in SH-SY5Y cells

Sample	LDH (% control)	SE	p-value	Viability (% control)	SE	p-value
Tris buffer (control)	100	2.4	NA	100	4.3	NA
αS	121.5	3.5	0.005^a^	67.9	1.9	0.004^a^
αS + curcumin	99.7	5.3	0.018^b^	89.1	4.1	0.008^b^
Curcumin	99.3	4.9	0.936	96.8	5.6	0.501

Cytotoxicity was measured using LDH assay and cell viability was determined using resazurin reduction assay. SH-SY5Y cells were incubated with Tris buffer (control), αS, αS+curcumin and curcumin for 48 hr before analysis. The LDH release and cell viability were expressed as a percentage of the untreated samples. Data was reported as mean ± SE, *n *= 4. ^a^compared with the control; ^b^compared with αS-treated samples.

### Extracellular addition of αS generates excessive ROS

**Figure 2:**
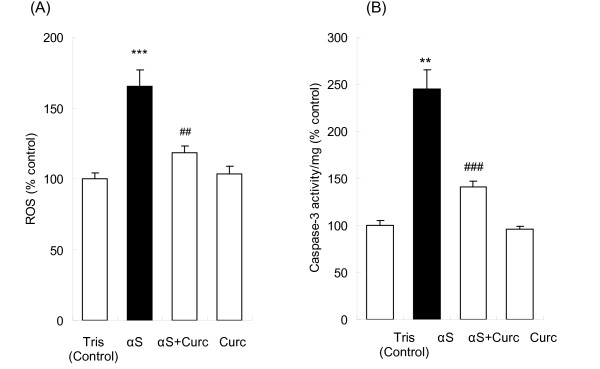
**Curcumin reduces αS-induced intracellular ROS generation and inhibits caspase-3 activation in SH-SY5Y cells**. SH-SY5Y cells were incubated with Tris buffer, oligomeric αS, αS+curcumin and curcumin and the intracellular ROS and caspase-3 activity were determined using cell based assays. (A) Intracellular ROS was determined by DCF fluorescence. DCFH-DA was then added to each well and the plate was incubated at 37°C for an additional 1 hr. The fluorescence intensity of DCF was measured at Ex_485 nm _and Em_535 nm_, respectively. The increase in DCF fluorescence was expressed as a percentage of the control and is a direct measurement of intracellular ROS due to the oxidation of DCFH-DA to DCF by intracellular ROS. (B) Caspase-3 activity was determined by the absorbance of pNA substrate. After 24 h of treatment, the cells were detached, lysed and an equal protein loading was added to the 2× reaction buffer with DTT and DEVD-pNA substrate. After 1 hr of incubation at 37°C, the absorbance intensity was measured at 405 nm and the caspase-3 activity was reported percentage of the Tris buffer control. Data was analyzed using one way ANOVA followed by Bonferroni post-hoc test and reported as mean ± SE, *n *= 4. **p < 0.01, ***p < 0.001, compared with the untreated samples; and ##p < 0.01, ##p < 0.001, compared with αS-treated samples

When oligomeric αS was added extracellularly to SH-SY5Y cells, the intracellular ROS level significantly increased from 100 ± 3.8 (control) to 165.4 ± 11.8 (Fig. 2A), indicating that extracellular αS enhances ROS levels in SH-SY5Y cells. Treatment with curcumin substantially reduces this increase in intracellular ROS levels to 118.5 ± 4.6 (Fig. 2A). Curcumin and Tris buffer alone did not affect ROS levels (Fig. 2A). The ability of curcumin to reduce ROS levels generated by oligomeric αS is consistent with results obtained using anti-oxidants in MPP^+ ^PD models [17,20,43] providing further evidence that ROS plays a central role in the selective degeneration of dopaminergic neurons in PD.

### Curcumin inhibits caspase-3 activity and apoptosis induced by extracellular αS

**Figure 3:**
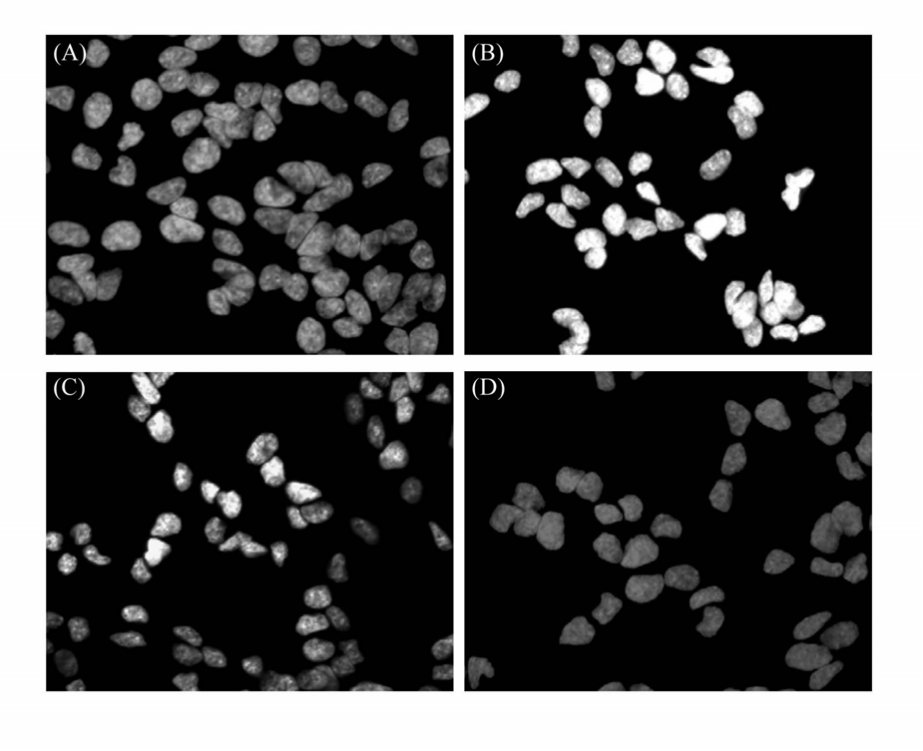
**Curcumin ameliorates αS-induced morphological changes in SH-SY5Y cells evaluated by fluorescence microscopy**. After 48 hr incubation, cells were fixed with 4% paraformaldehyde, stained with Hoechst 33342 (5 mg/mL) and analyzed using a Nikon TE300 fluorescence microscope. Fluorescence micrographs (100× magnification) of the (A) control cells, (B) cells exposed to αS, (C) cells exposed to αS+curcumin and (D) curcumin.

In addition to increasing ROS levels, extracellular incubation of SH-SY5Y cells with oligomeric αS also activates caspase-3 activity and triggers apoptosis. Caspase-3 activity in the oligomeric αS-treated sample increased by 2.4-fold compared to the control, while pre-incubation of curcumin with αS reduced the increase in caspase-3 activation by almost half (Fig. 2B). Addition of curcumin and Tris buffer alone had no significant effects on caspase-3 activity (Fig. 2B). Extracellular addition of oligomeric αS to SH-SY5Y cells also induced apoptosis in the cells as marked by the changes in morphologies of the cell nuclei. While the control SH-SY5Y cells had regular nuclei with uniformly dispersed chromatin and intact cell membrane (Fig. 3A), cells incubated with oligomeric αS showed signs of apoptosis as indicated by condensed nuclei and intense fluorescence staining with Hoechst dye (Fig. 3B). Pre-incubation of curcumin with αS reduced nuclear damage induced by extracellular oligomeric αS (Fig. 3C) while curcumin alone did not affect the nuclear morphology of the cells (Fig. 3D).

### Curcumin reduces ROS and cytotoxicity induced by intracellular overexpression of αS

**Figure 4:**
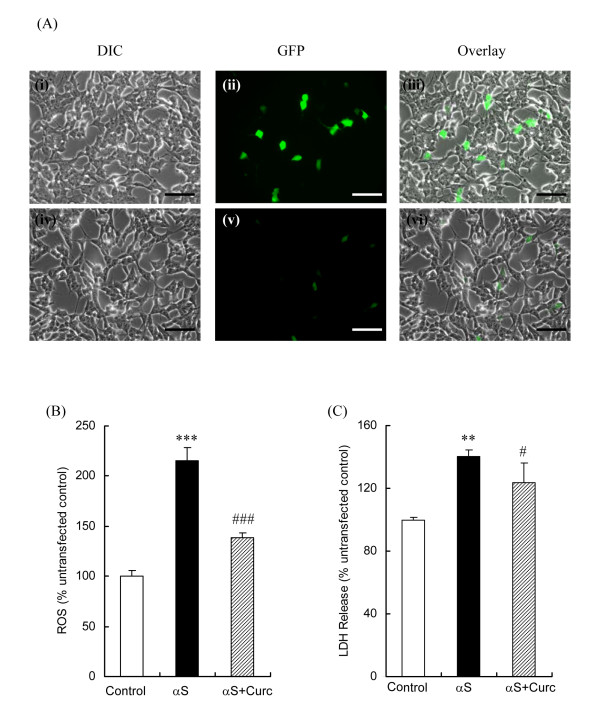
**Curcumin reduces intracellular ROS and cytotoxicity of transiently transfected WTαS-EGFP SH-SY5Y cells**. Localization of WTαS-EGFP and protective effects of curcumin in transiently transfected SH-SY5Y cells. (A) Representative images of WTαS-EGFP transfected cells in the absence (i-iii) and presence of curcumin (iv-vi) were captured using a Nikon TE300 fluorescence microscope. Scale bar = 50 μm. (B) ROS levels and (C) cytotoxicity of the untransfected control, αS-transfected and αS-transfected+curcumin were measured. For LDH assay, cell culture media was collected after 48 hr and the LDH release was expressed as a percentage of the untransfected samples. For ROS measurements, DCFH was added to each well and the DCF fluorescence was measured at Ex_485 nm _and Em_535 nm _after an additional incubation at 37°C for 30 min. DCF fluorescence was expressed as a percentage of the untransfected control reported as mean ± SD, *n *= 3. **p < 0.01; ***p < 0.001, compared with the untransfected control, #p < 0.05; ###p < 0.001 compared with αS-transfected sample analyzed using one way ANOVA followed by Bonferroni post-hoc test.

While curcumin provided substantial protection against extracellular αS-induced toxicity, PD pathology includes intracellular aggregation and accumulation of αS. We evaluated whether extracellularly added curcumin can also protect against intracellularly induced αS toxicity by overexpressing αS in SH-SY5Y cells by transient transfection with a WTsynEGFP gene. The αS-transfected cells showed intracellular eGFP fluorescence, indicating αS expression (Fig. 4A). The level of αS overexpression was estimated to be around 10% from 3 independent experiments, which was typical for this cell line [9]. The eGFP fluorescence intensity and the αS levels were markedly reduced in the curcumin treated sample, suggesting a suppressive effect of curcumin on αS expression (Fig. 4A). Intracellular ROS levels in the αS-transfected cells increased over 2-fold compared to untransfected cells, while treatment with curcumin reduced the increase in ROS level to just 40% over the control value (Fig. 4B). Similarly, intracellular αS increased LDH release by 40% compared to the control cells and addition of curcumin reduced the LDH increase to just 20% (Fig. 4C). The overexpression of αS significantly increased ROS and LDH levels and addition of curcumin alleviated these effects, even with the low expression levels studied. Curcumin therefore has similar potent protective effects against αS cytotoxicity regardless of whether αS toxicity is induced extra- or intracellularly.

## Discussion

A key pathological feature of PD is the formation of cytoplasmic inclusions containing ubiquitin and αS known as LBs and LNs in the dopaminergic neurons of the *substantia nigra *region of the brain [3,4]. The many factors that influence αS aggregation and the subsequent downstream cytotoxic events that lead to neuronal cell death are being actively studied. Several point mutations in the αS gene which correlate to rare familial early-onset PD and rapid progression of the disease [7-9] accelerate aggregation of αS and favor formation of nonfibrillar oligomeric forms. Recent studies have suggested that soluble oligomeric and protofibrillar structures are the toxic species [27-30], and that these forms can permeabilize plasma membranes, alter intracellular function, induce oxidative stress and trigger apoptosis in cells [30,44,45]. Epidemiological studies have also suggested that exposure to environmental agents such as neurotoxins and pesticides [16,17] cause an increase in oxidative damage to the cells by suppressing mitochondrial complex I activity and reducing glutathione levels [22,23], thereby increasing the risk for PD.

Oxidative stress plays a major role in aging and is associated with several neurodegenerative diseases including PD [46], where an increase in ROS accompanies αS aggregation and degeneration of dopaminergic neurons [15,47,48]. Intracellular overexpression of αS generates excess ROS and causes oxidative stress to the cells [23,46], leading to disruption in redox homeostasis cell metabolism, free radical generation, lipid peroxidation, cholesterol and protein oxidation [46,49]. Excess ROS causes plasma membrane damage, mitochondrial dysfunction, defects in the glutathione peroxidase expression and reduction in glutathione levels, all of which render the brain more susceptible to oxidative stress [46,49,50]. In this study, we find that extracellular addition of oligomeric αS and intracellular overexpression of αS in SH-SY5Y cells both increase ROS levels by almost 2-fold. The αS-induced increase in ROS levels in our current study shows similar oxidative damage to the SH-SY5Y cell as previous MPP^+ ^PD cell models, where MPP^+ ^selectively targets and degenerates dopaminergic neurons due to excess generation of ROS [13,15,18]. Prolonged exposure to MPP^+ ^and other neurotoxins has been shown to activate caspase-3 [16,19,51], an important effector caspase in the final apoptotic cascade leading to cell death. If oxidative stress exacerbates the etiology of PD, then agents that can simultaneously attenuate ROS damage and suppress caspase-3 activation may hold promise for the treatment of PD and other neurodegenerative diseases.

Here we show that curcumin, a natural phenolic food additive, effectively inhibits activation of caspase-3 (Fig. 2B) and ameliorates signs of apoptosis (Fig. 3) induced by extracellular addition of oligomeric αS to SH-SY5Y cells. We also demonstrated that curcumin reduces intracellular overexpression of αS and reduces ROS generation [15,46,48].

## Conclusions

Overexpression and abnormal accumulation of oligomeric αS is key in the pathogenesis of PD [14,48,52], and numerous studies suggest that there is both an intra- and extracellular component to αS toxicity in PD [12,24,31,32,53]. We recently demonstrated that an anti-oligomeric αS antibody fragment binds oligomeric αS on the surface of SH-SY5Y cells, verifying the presence of intracellularly produced oligomeric αS on external cell membrane surfaces [29]. Here we show that extracellular addition of oligomeric αS induces similar cytotoxic effects as intracellular overexpression of αS, and that these αS-induced cytotoxic effects are similar to those reported in MPTP Parkinsonian models. We also show that curcumin can significantly reduce the cytotoxicity induced by extracellular or intracellular αS aggregates, suggesting it may have value for treating PD. Since extracellularly added curcumin provides protection even against intracellularly induced αS toxicity, our results suggest that there is a significant extracellular or cell surface component of αS-induced toxicity in PD models, which is consistent with a recently published report of interneuronal transmission of extracellular αS pathology in neuronal cells [53]. However, additional studies are needed to further elucidate the mechanism of αS-induced cytotoxicity and its subsequent pathogenesis and progression to induced-apoptosis in PD.

## Methods

### α-synuclein aggregation

αS was prepared and purified in our lab as previously described [28,54]. Purified αS was lyophilized and stored at -80°C until further use. Stocks of the lyophillized αS were first dissolved in DI water and subsequent dilutions were made in Tris buffer (25 mM Tris, 150 mM NaCl, pH 7.4). The various forms of αS samples (70 μM) were prepared by dissolving the αS stock in Tris buffer. Monomeric αS samples were utilized immediately after dilution with Tris buffer, oligomeric αS were generated by incubating the samples at 37°C for 5-7 days (without shaking) while predominantly fibrillar morphologies of αS were generated by incubation at 37°C for up to 30 days (without shaking). αS morphologies were verified by AFM before use. All other chemicals were purchased from Sigma-Aldrich (Sigma-Aldrich, MO) and used as is without further treatment unless otherwise specified.

### Co-incubation of curcumin with pre-formed αS samples

Curcumin stocks (1 mg/mL) were prepared in dimethyl sulfoxane (DMSO) and stored at -20°C in dark conditions until use. Curcumin was diluted to 140 μM with Tris buffer in a 2:1 molar ratio of curcumin to pre-formed αS sample.

### Atomic Force Microscopy

A 10 uL aliquot of each sample was applied to a piece of freshly cleaved mica, incubated at room temperature for 10 minutes, rinsed with DI water and dried under a gentle stream of N_2 _gas. Topographic AFM images were acquired using OTESPA tips (k = 40 N/m, *f*_o _= 300-kHz) (Veeco, Santa Barbara, CA) at scan rates of 2 Hz with 512 × 512 pixel resolution on a Nanoscope IIIa TM-AFM (Veeco, Santa Barbara, CA). AFM images were analyzed with the scanning probe imaging processor software (SPIP, Image Metrology) to generate height distribution plots, as previously described [55].

### PAGE and silver staining

Oligomeric αS samples, with and without curcumin were separated on a 10% Tris/Tricine native PAGE and developed using Pierce silver stain kit according to manufacturer's protocol. (Thermo Scientific, Rockford, IL).

### Cell culture and transient transfection of SH-SY5Y cells

SH-SY5Y-human neuroblastoma cells were maintained and grown as described previously [28,29]. Transient transfection of SH-SY5Y cells was performed using TransFast™ transfection reagent according to the manufacturer's protocol (Promega, Madison, WI) with slight modification. SH-SY5Y cells were grown for 4 days (50-65% confluency) in a 6-well plate *in vitro *before transfection. A transfection mixture consisting of a 1 μg aliquot of wildtype α-synuclein/eGFP (WTsynEGFP) fusion protein plasmid DNA (Clontech, Palo Alto, CA) and TransFast™ reagent (1:2 v/v) in serum free media was pre-incubated in the dark for 15 min at room temperature before addition to the cells. Cell culture media was removed and the transfection mixture (500 μL) was added to each well and incubated for 1 hr at 37°C, followed by addition of complete media with serum (500 μL). The culture plates were incubated and grown in a 5% CO_2 _atmosphere at 37°C for 48 hrs. A 10 μL aliquot of curcumin (4 μM final concentration) was added 48 hr post-transfection and the cells were incubated for another 24 hr before analysis. Previous studies have shown that the α-synuclein fusion protein aggregates similarly to α-synuclein alone, and that eGFP expression does not induce toxicity [56].

### Cytotoxicity by lactate dehydrogenase assay

Cytotoxicity of samples towards SH-SY5Y cells was measured using a lactate dehydrogenase (LDH) assay as described [57]. Cells were seeded (2 × 10^4 ^cells/mL) in a 96-well plate 24 hr prior to the following treatment conditions: (a) pre-formed oligomeric αS (2 μM), (b) co-incubated samples of αS (2 μM) with curcumin (4 μM), (c) curcumin (4 μM) and (d) Tris buffer control. After incubating cells with each treatment for 48 hr, cytotoxicity of each sample was determined by measuring the reduction of iodonitrotetrazolium salt by LDH enzyme using a Wallac 1420 plate reader (Perkin Elmer, USA) at 490 nm and 650 nm. The values were expressed as a percentage of the Tris buffer control. Experiments were repeated a minimum of three times.

### Cell viability by resazurin reduction assay

Cell viability was determined using a resazurin reduction assay [58]. Viable cells convert resazurin (blue) to resorufin (pink), and the degree of cell death can be measured directly by either absorbance or fluorescence spectrometry. Resaruzin stocks (10 mM) were made in DMSO and kept at -20°C until use when they were diluted to a 100 μM working solution with Tris buffer. Cells were seeded (5 × 10^4 ^cells/mL) in a 48-well plate 24 hr prior to exposure to the treatment conditions described above. Following treatment for 48 hr, cell culture media was removed and the cells were resuspended with 200 μL Tris buffer. An aliquot (10 μL) of resaruzin (20 μM final concentration) was added to each well and incubated at 37°C for an additional 3 hr. Absorbance of resorufin was measured at 560 nm and 600 nm. Cell viability of each sample was calculated by subtracting the background OD_600 nm _from OD_560 nm _and reported as a percentage of the Tris buffer control.

### Measurement of intracellular ROS formation

The formation of intracellular ROS was measured using a fluorescent probe, 2,7-dichlorofluorescein diacetate (DCFH-DA) as described [59]. The cells were seeded (2 × 10^4 ^cells/mL) in a 96-well plate and were incubated for 48 hrs prior to ROS measurement with the conditions described above. After treatment, the cells were washed twice and resuspended in 100 μL Tris buffer. DCFH-DA (10 μM final concentration) was added to each well and the cells were incubated for 1 hr at 37°C in dark conditions. The fluorescence intensity of dichlorofluorescein (DCF, the oxidized species of DCFH-DA) was measured using a fluorescence spectrophotometer with excitation wavelength of 485 nm and emission wavelength of 535 nm.

### Determination of caspase-3 activity

Caspase-3 activity was determined using the Caspase-3/CPP32 colorimetric assay kit following the manufacturer's protocol (BioVision, Inc., CA). Since caspase-3 is a pre-apoptotic marker, measurements of caspase-3 activity were taken after 24 hr incubation with the various treatments to ensure proper detection. Briefly, cells (10^6 ^cells/mL) were exposed to different treatments as described above for 24 hr, detached and lysed on ice for 10 min. The supernatant was removed and the total protein concentration of each sample was determined using a bicinchoninic acid assay (BCA, Pierce, Rockford, IL). Cell lysate was then diluted to 150 μg with lysis buffer for each assay. An equal loading amount of lysate (50 μL) was mixed with 50 μL of 2× reaction buffer with 10 mM dithiothreitol (DTT) and 5 μL DEVD-pNA substrate (200 μM) and incubated at 37°C for 1 hr. The absorbance of released *p*-nitroanilide (*p*NA) was measured at 405 nm using a plate reader. The increase in caspase-3 activity was determined by comparing the absorbance of the treated sample with the absorbance of the Tris buffer control sample.

### Fluorescence microscopy and nuclear staining

WTsynEGFP-transfected cells were evaluated 48 hr post-transfection using a Nikon TE300 fluorescence microscope at an excitation wavelength of 488 nm with a 40× magnification objective. For nuclear staining, untransfected SH-SY5Y cells were seeded on glass coverslips and allowed to attach for 24 hr. The cells were fixed with 4% paraformaldehyde for 25 min, washed twice in cold Tris buffer, and stained with Hoechst 33342 (10 μg/mL) for 15 min. Nuclear morphology was observed using a 100× magnification objective. Images were captured and processed by MetaMorph software (Molecular Devices, USA). Cells stained by Hoechst 33342 with diffused nuclei were scored as viable, while cells with reduced nuclei, condensed chromatin, and increased fluorescence were considered apoptotic.

### Statistical Analysis

Data was presented as mean ± SE from at least three independent experiments. Statistical analysis was evaluated using either Student's t-test or using a one-way ANOVA followed by Bonferoni post-hoc test for all pair-wise comparison. A p-value of < 0.05 was considered as significant.

## Authors' contributions

MSW participated in the experimental design of the study, prepared pre-aggregated αS samples, conducted in vitro cell-based assays, performed fluorescence microscopy and statistical analysis and drafted the manuscript. SB maintained the untransfected SH-SY5Y cells, plated and added samples for all the in vitro assays and edited the manuscript. SE conducted transient transfection of SH-SY5Y cells and edited the manuscript. MRS supervised the design of study, provided technical assistance in data interpretation, and played a major part in revision of the manuscript. All authors read and approved the final manuscript.

## Supplementary Material

Additional file 1 **Curcumin alters αS aggregation kinetics**. Aggregation kinetics were monitored using Thioflavin T assay. (A) monomeric αS and monomeric αS + curcumin, (B) pre-formed oligomeric αS and pre-formed oligomeric αS + curcumin, (C) pre-formed fibrillar αS and pre-formed fibrillar αS + curcumin. Samples were taken at the indicated time and mixed with Thioflavin T. The observed Thioflavin T fluorescence (Ex = 450 nm, Em = 482 nm) was normalized to each untreated αS sample at its maximum value. Data are presented as mean ± SE from 4 sets of experiments. Click here for file
