# Supplementary figures and images for: Curcumin reduces α-synuclein induced cytotoxicity in Parkinson's disease cell model
Source: BMC Neurosci. 2010 Apr 30;11:57. doi: 10.1186/1471-2202-11-57 (PMC2879277; doi:10.1186/1471-2202-11-57)

(A)

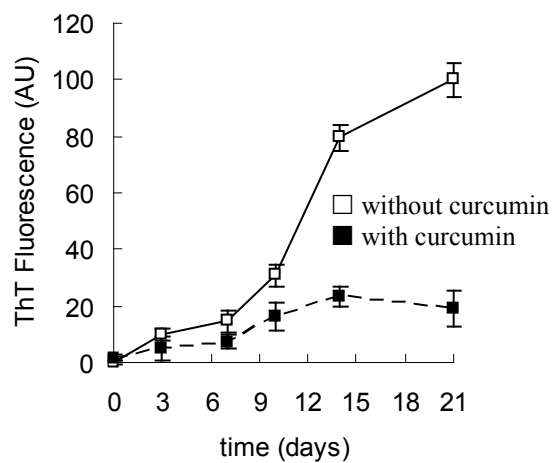

(B)

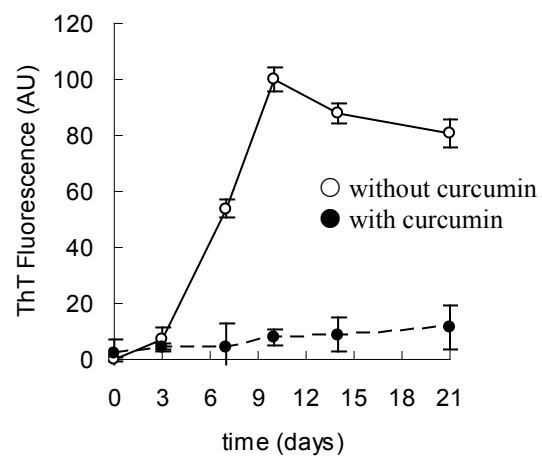

(C)

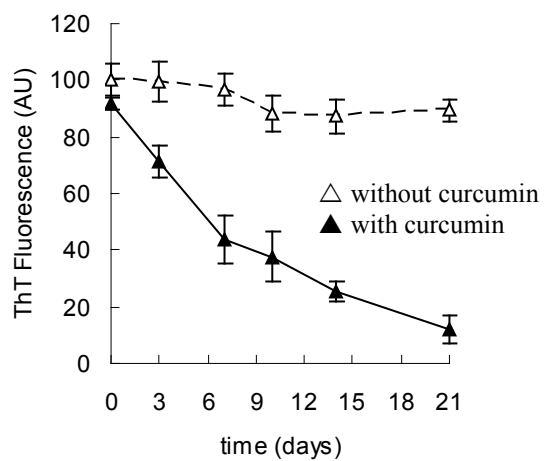

Supplement: Additional file 1 — Curcumin alters αS aggregation kinetics. Aggregation kinetics were monitored using Thioflavin T assay. (A) monomeric αS and monomeric αS + curcumin, (B) pre-formed oligomeric αS and pre-formed oligomeric αS + curcumin, (C) pre-formed fibrillar αS and pre-formed fibrillar αS + curcumin. Samples were taken at the indicated time and mixed with Thioflavin T. The observed Thioflavin T fluorescence (Ex = 450 nm, Em = 482 nm) was normalized to each untreated αS sample at its maximum value. Data are presented as mean ± SE from 4 sets of experiments. [file 1471-2202-11-57-S1.PDF]
